# Effect of Glucans from *Caripia montagnei* Mushroom on TNBS-Induced Colitis

**DOI:** 10.3390/ijms15022368

**Published:** 2014-02-10

**Authors:** Marilia S. da Nascimento Santos, Joedyson Emmanuel M. de Magalhães, Luiza Sheyla Evenni P. Will Castro, Thuane de Sousa Pinheiro, Diego Araujo Sabry, Leonardo Thiago Duarte B. Nobre, João Paulo Matos Santos Lima, Iuri Goulart Baseia, Edda Lisboa Leite

**Affiliations:** 1 Laboratory of Glycobiology, Department of Biochemistry, Federal University of Rio Grande do Norte, Av. Salgado Filho, 3000, Bairro L. Nova, CEP 59078-970, Natal, RN, Brazil; E-Mails: mariliabio84@gmail.com (M.S.N.S.); joedyson_magalhaes@hotmail.com (J.E.M.M.); lsepwill_@gmail.com (L.S.E.P.W.C.); tutu_sousa@hotmail.com (T.S.P.); leo.dnobre@gmail.com (L.T.D.B.N.); jplslima@gmail.com (J.P.M.S.L.); 2 Department of Biochemistry, Federal University of Paraná, UFPR, Curitiba CEP 81531-980, PR, Brazil; E-Mail: popoh_diego@hotmail.com; 3 Laboratory of Mycology, Department of Botany, Zoology and Ecology, Federal University of Rio Grande do Norte, Av. Salgado Filho, 3000, Bairro L. Nova 59078-970, Natal, RN, Brazil; E-Mail: baseia@cb.ufrn.br

**Keywords:** colitis, polysaccharides, mushroom, anti-inflammatory, *Caripia montagnei*

## Abstract

In this study, we evaluated the effect of different doses of polysaccharides extracted from *Caripia montagnei* mushroom at different intervals of treatment on colonic injury in the model of colitis induced by 2,4,6-trinitrobenzene sulfonic acid (TNBS). The FT-IR analysis and NMR showed that the polysaccharides from this species of mushroom are composed of α- and β-glucans. The colonic damage was evaluated by macroscopic, histological, biochemical and immunologic analyses. The results showed the reduction of colonic lesions in all groups treated with the glucans. Such glucans significantly reduced the levels of IL-6 (50 and 75 mg/kg, *p* < 0.05), a major inflammatory cytokine. Biochemical analyses showed that the glucans from *C. montagnei* acted on reducing levels of alkaline phosphatase (75 mg/kg, *p* < 0.01) and myeloperoxidase (*p* < 0.001), a result confirmed by the reduction of cellular infiltration observed microscopically. The increase of catalase activity possibly indicates a protective effect of these glucans on colonic tissue, confirming their anti-inflammatory potential.

## 1. Introduction

Ulcerative colitis, which together with Crohn’s disease includes inflammatory bowel diseases (IBDs), consists of an idiopathic inflammatory process involving the mucosa of the colon and rectum, whose incidence varies between populations [1,2].

The pathogenesis of chronic intestinal inflammation results from an intestinal mucosa dysfunction resulting from the overproduction of pro-inflammatory mediators that trigger the immune system alterations. Allied to this is the suggestion that the union of genetic factors confers disease susceptibility to environmental factors [3].

Clinical manifestations are characterized by changes in gastric motility, weight loss, ulceration of the colon mucosa, fever, shortening of the colon and diarrhea with blood and/or mucus [4].

Current treatments include commonly used drugs, such as aminosalicylates, which assist in maintaining remission of crises, corticosteroids, which are utilized during acute episodes, and immunomodulators [5]. However, these treatments are often associated with severe side effects and high costs [6,7]. Thus, there is a search for safe natural compounds that can contribute to the prevention or even treatment of inflammatory diseases [8–11].

Initial studies with the aqueous extract of *Caripia montagnei* mushroom found its anti-inflammatory potential. In this study, the polysaccharidic extract was able to not only reduce inflammatory edema and reduce levels of leukocyte migration, but also significantly reduce the levels of cytokines and nitric oxide [10]. The aim of this study was to evaluate the action of glucans from the *Caripia montagnei* model on 2,4,6-trinitrobenzene sulfonic acid (TNBS)-induced colitis in rats and their effects on interleukin levels (IL-1 IL-6), catalase, myeloperoxidase (MPO) enzymes and nitric oxide. Moreover, assessment of their action in colonic tissue was performed by histological analysis.

## 2. Results

### 2.1. Chemical Analysis

We previously reported that the aqueous extract rich in polysaccharides of the fruiting bodies of *Caripia montagnei* showed anti-inflammatory potential using the models, carrageenan-induced plantar edema and peritonitis induced by thioglycollate. There was a reduction in inflammatory edema and in the levels of leukocyte migration, lowering the levels of nitric oxide and cytokines, in addition to inhibiting expression of nuclear factor κB peritoneal lavage from mice [10]. The aqueous extraction of polysaccharides is a technique widely used in scientific studies [12–14]. Its efficiency in obtaining polysaccharides, high yield and low expense may be the main reasons for their large employment.

The measurements revealed that the compound extracted from the mushroom, *Caripia montagnei*, is composed mainly of polysaccharides (96%), had a low protein content (2.5%) and phenolic compounds (1.5%). The monosaccharide composition showed the glucose as sugar predominantly existing in the obtained polysaccharide fraction of *C. montagnei* [10].

### 2.2. FT-IR Spectrum

**Figure 1.**
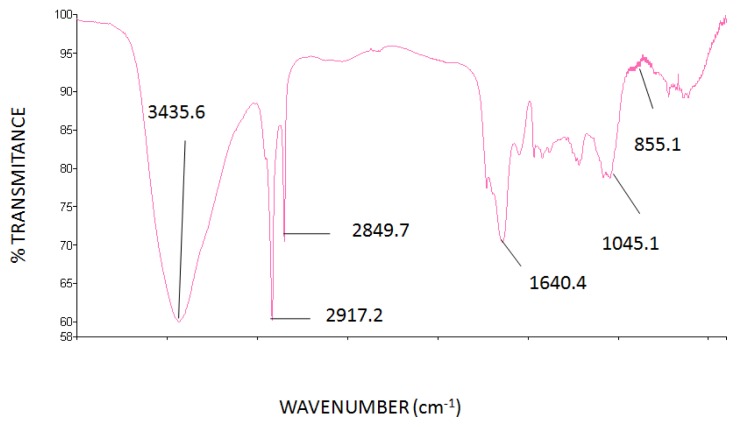
FT-IR spectra of the polysaccharides obtained by aqueous extraction followed by ethanol precipitation of the *Caripia montagnei* mushroom.

The FT-IR analysis (Figure 1) revealed the broadband centered around 3500 cm^−1^, which is a characteristic sign of the band of axial stretching of OH. The existing signal in the range of 2900 and 2800 cm^−1^ means the presence of aldehyde grouping (CH) in axial strain. The analysis also revealed absorptions around 1300–1800 cm^−1^, which are characteristic of the presence of carbohydrates [15,16]. Moreover, the analysis revealed the presence of the band between 1000 and 1100 cm^−1^, *i.e.*, 1045 cm^−1^ being characteristic of the presence of β-glucans due to *O*-substituted glucose residues [17,18]. A signal was found in the range of 855 cm^−1^, which corresponds to the presence of α-glucans [19].

### 2.3. NMR

**Figure 2.**
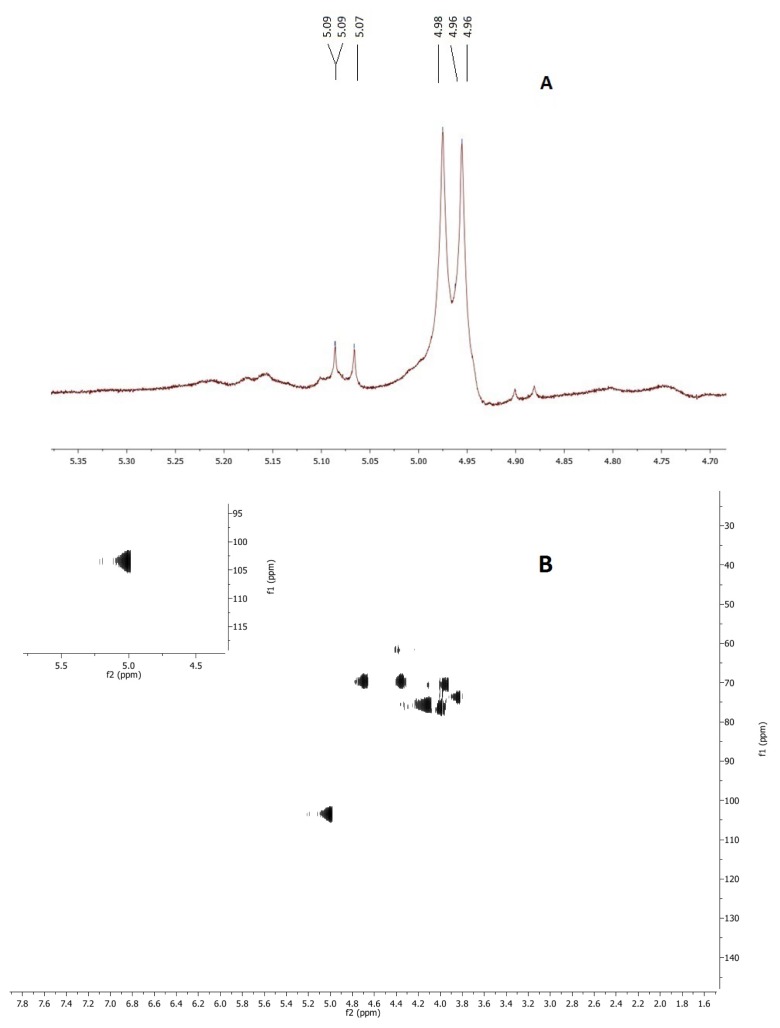
Chemical characterization of the polysaccharides of *Caripia montagnei.* (**A**) NMR spectrum of ^1^H; (**B**) HSQC spectrum solution in D_2_O.

The ^1^H spectrum of polysaccharides from *C. montagnei* showed the anomeric signals in the region 4–5 ppm (Figure 2A). The signs existing in 5.07 and 5.9 ppm are attributed to the presence of α-glucans. Signs existing in 4.96 and 4.98 ppm correspond to the presence of β-glucans [20], corroborating the results found in the analysis of FT-IR. The main anomeric signals (C-1/H-1) in the “heteronuclear single quantum correlation spectroscopy” (HSQC) (Figure 2B) were up and 100.55/5.01, 103.32/5.01 and 103.99/4.97, corresponding to the C-1 units A, B and C, respectively, in accordance with the decrease in anomeric chemical shifts [21]. The signals at low resonance frequency in C-1 to 100.32 and 103.55 and at high frequency in H-1 at 5.01 indicate the presence of →α-d-glc→ [22–24], while the signal at 103.99/4.97 indicates the presence of terminal Glc (β-Glc H-1 to 4.97 ppm).

### 2.4. Macroscopic Analysis

Trinitrobenzene sulfonic acid (TNBS) and dextran sulfate sodium (DSS) or oxazolone are typically used in models of inflammatory bowel disease (IBD) chemically-induced inflammation, due to the immediate, high reproducibility and simplicity of the induction process [25].

**Figure 3.**
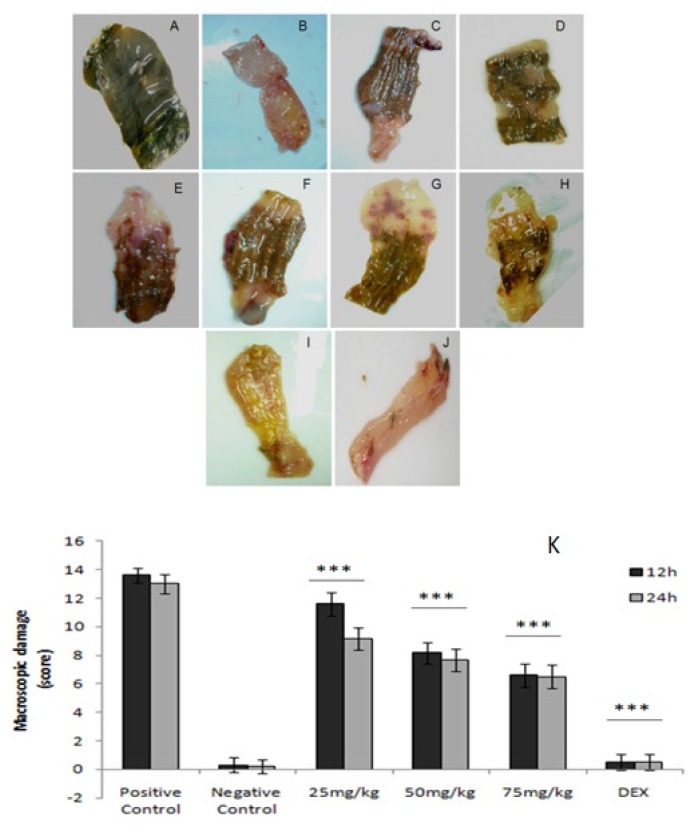
Macroscopic colonic lesions of rats (*n* = 3) with 2,4,6-trinitrobenzene sulfonic acid (TNBS)-induced colitis. (**A**) Untreated animals: positive control; (**B**) a negative control; (**C**,**D**) treated every 12 and 24 h, respectively, with 25 mg/kg of glucans of *Caripia montagnei*; (**E**,**F**) treated at intervals of 12 and 24 h, respectively, with 50 mg/kg of glucans of *C. montagnei*; (**G**,**H**) treated every 12 and 24 h, respectively, with 75 mg/kg of glucans of *C. montagnei*; (**I**,**J**) treated every 12 and 24 h, respectively, with 100 mg/kg dexamethasone; (**K**) the effect of glucans of *Caripia montagnei* in colonic inflammation. Data are expressed as the mean ± standard deviation. *** *p* < 0.001.

Glucans extracted from *Caripia montagnei* mushroom were used to evaluate their anti-inflammatory potential that is well described in a model of colitis induced by TNBS. After the induction of colitis, the presence of colonic lesions, such as hyperemia and ulcerations, was observed. As can be seen in Figure 3, the intracolonic administration of TNBS promoted a considerable increase of macroscopic lesions. In the positive control group were commonly found lesions, such as ulcers and necrosis (Figure 3A), in contrast to the negative control group in which there were no injuries (Figure 3B). Treatment of animals with dexamethasone (Figure 3I,J) and glucans from *C. montagnei* at different intervals and doses was able to reduce the damage observed (Figure 3C–H).

Macroscopic analyses were evaluated for the intestinal damage represented in Figure 3A–H by assigning a score and showed the extent and severity of intestinal damage. In this analysis, the score, which shows the severity of intestinal damage, indicated that the untreated group, the positive control, presented a high score (13.6 ± 0.51), showing that the animal’s intestinal injury induced colonic inflammation (Figure 3K). A significant reduction could also be seen between the positive control group and the group treated with dexamethasone (0.5 ± 0.54 for both time intervals) and the groups treated with glucan to 75 mg/kg at 12 h intervals (6.6 ± 0.81) and 24 h (6.5 ± 0.83).

### 2.5. Activity of Myeloperoxidase (MPO)

Among the biochemical parameters studied here, the changes in the myeloperoxidase (MPO), alkaline phosphatase (ALP) and catalase (CAT) activities are highlighted.

MPO is an enzyme found in the azurophilic granules of neutrophils, and therefore, their quantification is related to the presence of these cells in different tissues, including the gastrointestinal tract. It was suggested that the influx of neutrophils at active sites of inflammation governs the process of various inflammatory diseases [26]. Activated neutrophils produce reactive species of oxygen and nitrogen in the intestinal mucosa, inducing oxidative stress, which plays a significant role in the pathogenesis of inflammatory bowel disease [27,28].

**Figure 4.**
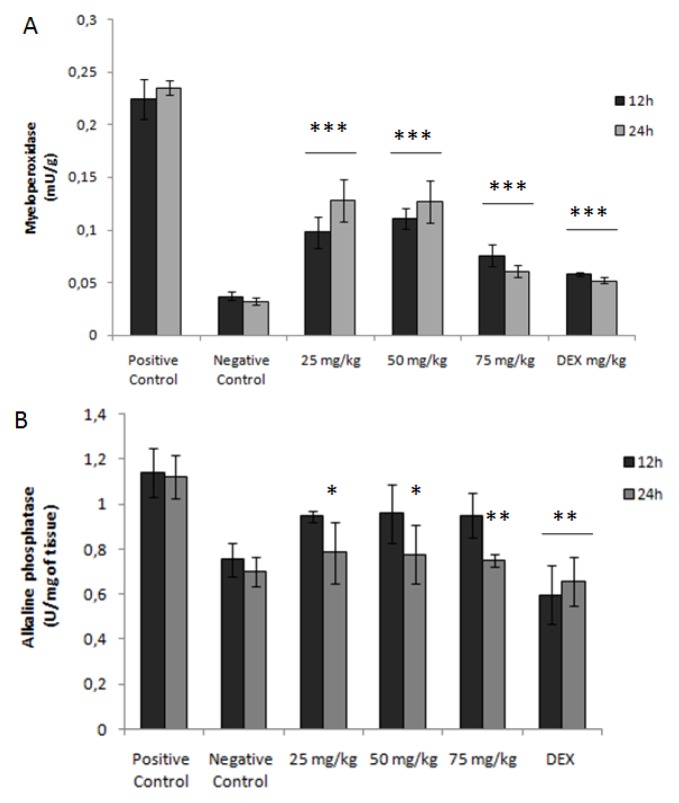
Evaluation of the influence of the glucans from *Caripia montagnei* on enzyme activity. (**A**) Myeloperoxidase and (**B**) alkaline phosphatase in colonic tissue (*n* = 4) with TNBS-induced colitis. Data were expressed as the mean ± standard deviation. * *p* < 0.05; ** *p* < 0.01; *** *p* < 0.001.

The activity of the enzyme, myeloperoxidase (MPO), was evaluated as a parameter to check the anti-inflammatory activity of the glucans of *C. montagnei.* Figure 4A shows that the administration of TNBS increased the MPO activity by more than six times when compared to the negative control. The results also showed that the treatments performed at different intervals (24/24 h, 12/12 h) with varying doses of glucans of *C. montagnei* were able to reduce the enzymatic activity significantly. The dose of 75 mg/kg glucans reduced the MPO activity by about 3.7 and 3.8 times (*p* < 0.001) when administered every 12 and 24 h, respectively. The reduction in enzyme activity was dose-dependent, and different ranges of treatments showed no statistically significantly interference in the activity of the glucans of *C. montagnei*.

### 2.6. Activity of Alkaline Phosphatase

The activity of the enzyme, alkaline phosphatase (AP), was another colonic biochemical parameter analyzed, and several studies demonstrate the upregulation of alkaline phosphatase and the mechanisms involved in experimental colitis [29]. High levels (1.14 ± 0.011 and 1.21 ± 0.097 U/mg for the groups treated every 12 and 24 h, respectively) of AP were found in groups with TNBS-induced colitis (Figure 4B). However, the reason for the increased colonic AP found in ulcerative colitis is unknown, and it was previously reported that AP is a sensitive and safe marker of experimental colitis in mice [29]. The activity of the colonic enzyme, alkaline phosphatase (AP), was significantly reduced in the groups treated with 50 mg/kg (0.77 ± 0.13 U/mg; *p* < 0.05) and 75 mg/kg (0.75 ± 0.028 U/mg; *p* < 0.01) glucans from *C. montagnei* at intervals of 24 h. The observed effect was dose-dependent, and a reduction of up to 33% ± 2.5% in the activity of AP was observed.

### 2.7. Nitric Oxide

The increased generation of free radicals was also considered as having a key role in the pathogenesis of IBD [30,31]. During inflammation, monocytes are being recruited into the parenchyma tissue, which are activated to make cells with a phagocytic function [32]. These cells release cytokines, free radicals and nitric oxide, which can mediate tissue injury related to the inflammatory response [33,34].

**Figure 5.**
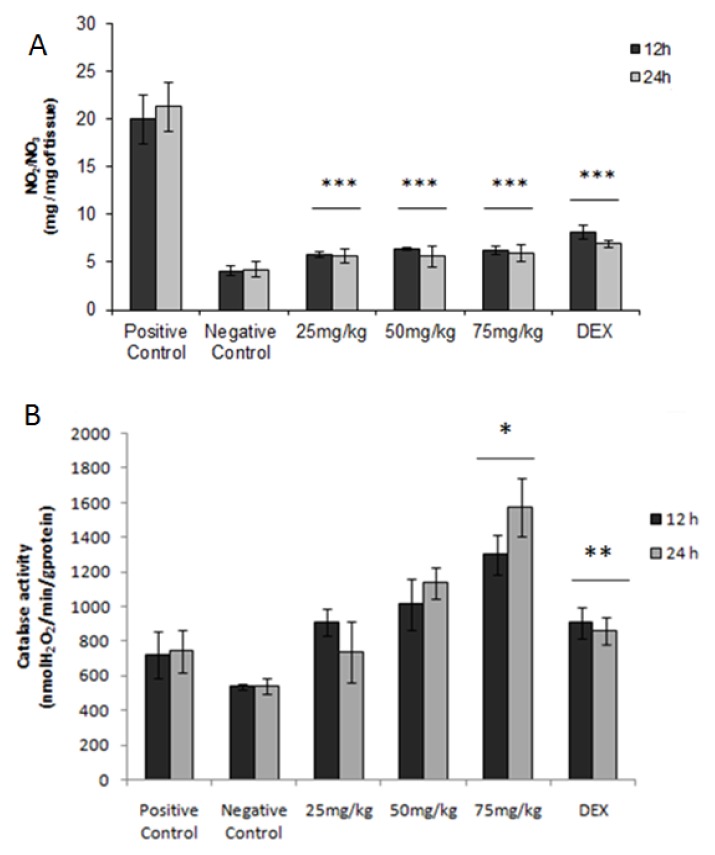
(**A**) The effect of different doses of the glucans from *Caripia montagnei* in NO content in colonic tissue and (**B**) catalase in colonic inflammation in the model of TNBS-induced colitis. Data were expressed as the mean ± standard deviation. * *p* < 0.05; ** *p* < 0.01; *** *p* < 0.001 was considered statistically significant.

Nitric oxide in most body fluids is rapidly metabolized to stable products, such as nitrite and nitrate. According to the results obtained (Figure 5A), the glucans from *C. montagnei* showed a high reduction (*p* < 0.001) of NO_2_/NO_3_ content in all the groups (*n* = 4) treated with the glucans at intervals of 12 and 24 h. In inflammatory reactions, NO-derived cells stimulated by the action of cytokines are involved with changes in the vascular permeability of the inflamed tissue [35]. Thus, it is possible that the reduction of inflammatory mediator NO relates to the anti-inflammatory potential of the polysaccharides of *Caripia montagnei*.

### 2.8. Activity of Catalase

The increased production of free radicals and a low concentration of endogenous antioxidant defense are related to damage to the intestinal mucosa in inflammatory bowel diseases [36]. Oxidative stress is considered a potential etiological factor for Crohn’s disease [37]. Then, the antioxidant system is usually used to evaluate the protective effect of various compounds in colonic inflammation. The levels of catalase in the colonic region of the group of animals (*n* = 4) that did not receive TNBS were 537.23 ± 17.05 nmol H_2_O_2_/min/g protein. The administration of TNBS increased the levels of CAT to 723.52 ± 137 nmol H_2_O_2_/min/g protein. In all groups treated with the glucans from *C. montagnei*, a moderate increase was observed in CAT levels in a dose-dependent manner up to 1300.61 ± 151 (*p* < 0.05) and 1577.28 ± 170 (*p* < 0.05) in the groups treated with 75 mg/kg at intervals of 12 and 24 h, respectively (Figure 5B).

### 2.9. Cytokine Analysis

**Figure 6.**
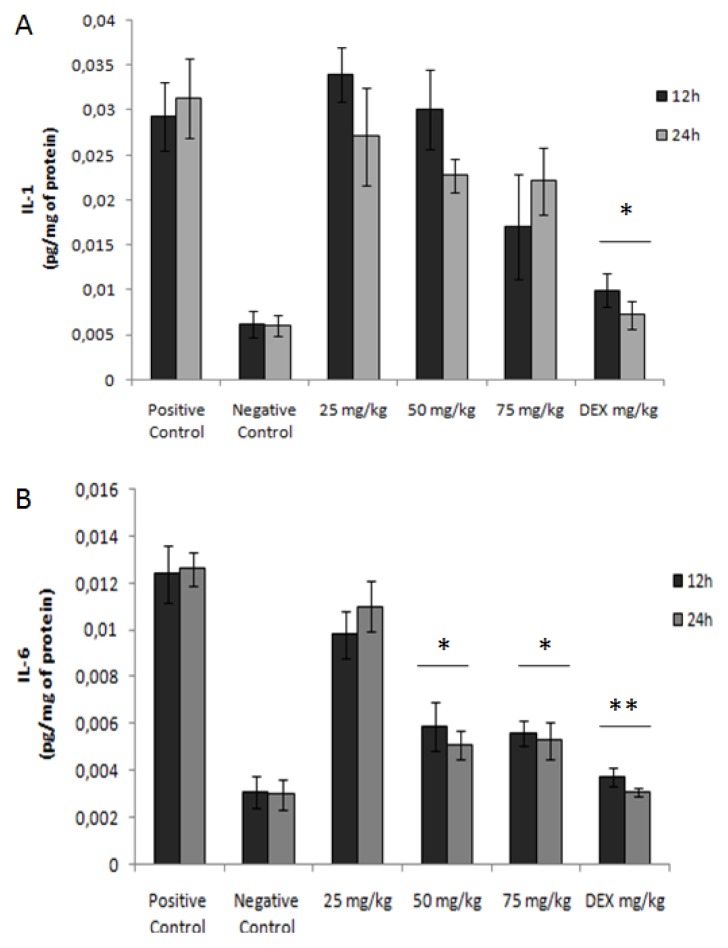
The effect of glucans from *Caripia montagnei* in modulating the release of cytokines (**A**) IL-1 and (**B**) IL-6 in colonic tissue (*n* = 4) with inflammation induced by TNBS. Data were expressed as the mean ± standard deviation. * *p* < 0.05; ** *p* < 0.01.

The effects of the derivatives of mushrooms include mitogenesis and activation of immune cells, such as hematopoietic cells, lymphocytes, macrophages and NK cells, resulting in cytokine production [38]. The environmental changes observed in colonic intestinal inflammation are usually associated with atypical immune responses of T-cells, which often lead to changing levels of cytokines. Glucans from *Caripia montagnei* were not able to alter the levels of IL-1 in all groups tested (Figure 6A). However, also verified was the effect of the glucans from *C. montagnei* on the release of IL-6-treated groups at different intervals and in a dose-dependent decrease, even reaching 64.8% ± 4.11% and 57.2% ± 6.45% of the cytokine levels in the colonic tissue of the group treated with 75 mg/kg glucan in intervals of 12 and 24 h, respectively (Figure 6B). Among the substances most commonly used in the treatment of inflammatory bowel disease is tocilizumab, which operates on the inhibition of IL-6 [39]. Thus, glucans from *C. montagnei* also showed potential to reduce the levels of this cytokine and nitric oxide in colonic tissue.

### 2.10. Histological Analyses

**Figure 7.**
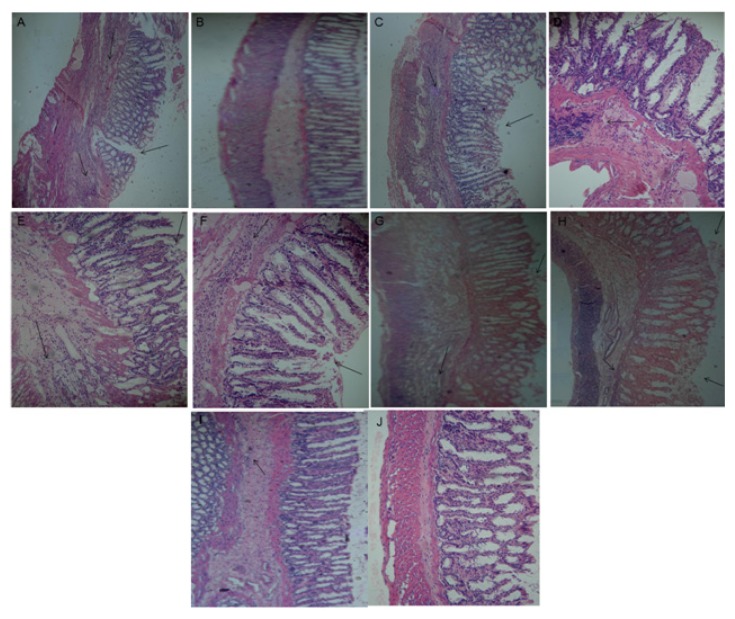
Histological analysis of colon of different groups of animals with TNBS-induced colitis. (**A**) untreated animals: positive control; (**B**) a negative control; (**C**,**D**) treated every 12 and 24 h, respectively, with 25 mg/kg of glucans of *Caripia montagnei*; (**E**,**F**) treated at intervals of 12 and 24 h, respectively, with 50 mg/kg glucans of *C. montagnei*; (**G**,**H**) treated every 12 and 24 h, respectively, with 75 mg/kg of glucans of *C. montagnei*; (**I**,**J**) treated every 12 and 24 h, respectively, with 100 mg/kg of dexamethasone. The figures with arrows indicate destruction of the mucosal layer with infiltration of inflammatory cells.

The high neutrophil infiltration is a consequence very characteristic of this experimental model of acute inflammation. Histologic examination revealed the destruction of the mucosal layer with prominent infiltration of inflammatory cells in the submucosa (Figure 7A). The negative control group showed no morphological tissue changes (Figure 7B). The treatment with dexamethasone (Figure 7I,J) and the glucans from *C. montagnei* at different intervals of time and dosage (Figure 7C–H) were able to reduce infiltration, corroborated with the observed reduction in the activity of the myeloperoxidase enzyme. The levels of tissue MPO activity are used as a quantitative measure for the infiltration of neutrophils in the inflammatory response in both clinical and experimental studies [40]. In this study, histological analyses confirm the reduction of MPO levels in groups treated with polysaccharides of *C. montagnei*, which can be viewed as reducing the cellular infiltration in the inflamed tissue.

## 3. Discussion

Mushrooms appear to be a good source of natural products [41,42], especially anti-inflammatory ones [43,44]. Aqueous extracts from mushrooms have long been used in oriental medicine as natural drugs. In addition, several studies have demonstrated the pharmacological potential of these extracts [45–47]. Fungal polysaccharides have severe effects on a variety of leukocytes, including macrophages, neutrophils, eosinophils and NK cells, as well as non-immune cells, such as endothelial cells, alveolar epithelial cells and fibroblasts [48–50].

Inflammatory bowel disease is complex, involving a wide range of molecules, including cytokines. Recent investigations support the important role of interleukin-6 (IL-6) in inflammatory bowel disease, showing that these cytokine levels are increased in patients with this inflammatory process [51]. NF-κB is a transcriptional factor that regulates the synthesis of cytokines (TNF-α, IL-1β, IL-6 and IL-8). In addition, it can stimulate iNOS, increasing the production of NO [52–54]. Mushrooms have been shown to possess anti-inflammatory activity via the suppression of the expression of interleukins and NO [55,56]. Previous studies with extracts of *C. montagnei* showed that these polysaccharides were able to reduce inflammation in a model of acute peritonitis reducing the expression of NF-κB [10]. In the present study, *Caripia montagnei* was shown to reduce the levels of important mediators, such as the cytokines, IL-6 and NO. In inflammatory reactions, NO-derived cells stimulated by the action of cytokines are involved with changes in the vascular permeability of the inflamed tissue. The decreased levels of NO were reported to be a key factor in reducing inflammation. The anti-inflammatory activity of the mushroom, *I. obliquus*, was attributed to the inhibition of the production of this inflammatory mediator [57]. In the model of colitis induced by TNBS, polysaccharides of *C. montagnei* showed reduced levels of IL-6 and NO, as well as others important markers, such as myeloperoxidase. Catalase (CAT) is an important cellular antioxidant. CAT is able to degrade hydrogen peroxide to form water. The protective effect of *C. montagnei* verified by increased levels of catalase may be associated with the reduced generation of reactive oxygen species. Thus, the anti-inflammatory activity displayed by these polysaccharides can be attributed to the inhibition of these important inflammatory mediators.

## 4. Experimental Section

### 4.1. Mushrooms

The mushrooms were collected in areas of the Atlantic Forest in the city of Natal (Rio Grande do Norte, Brazil), and after collection, the fungi were identified by Prof. Dr. Iuri Goulart Baseia, PhD in the Department of Zoology, Botany and Ecology, UFRN. The species were deposited in the herbarium at UFRN (UFRN fungi-836).

### 4.2. Animals

The studies were conducted with male *Wistar* rats (150–200 g) kept in a vivarium of the Department of Biochemistry, UFRN. All animals used in the testing were subjected to food and water *ad libitum* in controlled light conditions (12 h light/dark) and a temperature constant at 23 ± 2 °C. The animals were acclimatized in the laboratory for at least 4 h before the experiments and were used only once. The tests were approved (n° 014/2010) by the Ethics Committee of the Federal University of Rio Grande do Norte (UFRN) and followed the established norms.

### 4.3. Materials

Glycine, glucose, galactose, arabinose, fucose, mannose, rhamnose, glucuronic acid and xylose gallic acid, 3,3′,5,5′-tetramethylbenzidine, ethylenediaminetetraacetic acid, hexadecyltrimethylammonium bromide and 2,4,6-trinitrobenzene sulfonic acid were purchased from Sigma (St. Louis, MO, USA) and lyophilized. IL-1 and IL-6 were purchased from BD Pharmingen (San Diego, CA, USA). D_2_O, Folin Ciocalteau and KBr were purchased from Sigma (St. Louis, MO, USA).

### 4.4. Polysaccharide Extraction and Fractionation

The attainment of polysaccharides from *C. montagnei* was performed by modifications to the methodology proposed [10]. To obtain the polysaccharides from the fruiting bodies of the mushrooms, they were washed and dried at 40 °C and then were sprayed. For the extraction of the polysaccharides, 50 g of the tissue of the mushroom were used. To this powder was added 2 volumes of 80% acetone, leaving the mixture at 25 °C for 24 h and then filtering it. This was followed by extraction with chloroform-methanol (2:1, *v*/*v*) for 2 h at 60 °C under reflux and then filtering it. The supernatant was discarded, and to the precipitate was added 500 mL of distilled water, and it remained in the mixing bath at 100 °C for 3 h. The supernatant was treated with ethanol (3:1, *v*/*v*), resulting in a precipitate that was separated from the supernatant by centrifugation (4000× *g* for 20 min at 25 °C). Next, the obtained precipitate was dried and pulverized.

### 4.5. Chemical Analysis

Total sugars were determined using phenol/sulfuric acid, as previously described, employing l-glucose as the standard, with readings taken at 490 nm [58]. The protein content was determined with the Coomassie blue reagent with readings taken at 595 nm [59]. The concentration of total phenolics was determined colorimetrically according to the standard procedure of Folin-Ciocalteu [60], and the readings were taken at 755 nm. Aqueous solutions of gallic acid were used for the calibration curve. The total phenols, sugars and proteins were determined by interpolating the absorbance of the sample against the corresponding calibration curve.

### 4.6. Infrared Analysis

Infrared spectroscopy was performed on a 65 FT-IR PERKIN ELMER 104 spectrometer (PerkinElmer Inc., Wellesley, MA, USA), from 4000 to 400 cm^−1^. The polysaccharide was examined after drying under the form of a KBr wafer.

### 4.7. Nuclear Magnetic Resonance (NMR)

NMR analyses were performed at Universidade Federal do Paraná (UFPR) in a Bruker magneto model DRX 400 AVANCE series (Bruker BioSpin GmbH, Rheinstetten, Germany), in a pipe (wide-bore probe) of a 5-mm external diameter. The spectra were obtained at 80 °C using 10 mg of polysaccharides dissolved in 0.5 mL of deuterated water (D_2_O) (99.75%).

### 4.8. Anti-Inflammatory Activity in the Ulcerative Colitis Model Induced by 2,4,6-Trinitrobenzene Sulfonic Acid (TNBS)

The experimental colitis is a well-established model of intestinal inflammation and was induced in male rats (*n* = 10) as previously described [61]. Animals were fasted for 24 h before the experiment with free access to 5% glucose solution and were anesthetized with intraperitoneal administration of ketamine (80 mg/kg) and xylazine (10 mg/kg). The induction of colitis was conducted by intracolonic administration of 30 mg of 2,4,6-trinitrobenzene sulfonic acid (TNBS) after being lyophilized, in 0.25 mL of 40% ethanol (*v*/*v*) via a polyethylene catheter inserted into the lumen of the colon. This same procedure was carried out in a negative control group. However, control animals received 0.25 mL saline (0.9%).

### 4.9. Treatment of Colitis with Polysaccharides

The rats were divided into two experimental groups: (1) the control group rats, both negative and positive, received only saline treatment; (2) all other mice in the other groups were treated with different doses of polysaccharides (25, 50 and 75 mg/kg) dissolved in saline. Treatments were administered intraperitoneally 24 h after induction of experimental colitis and were performed in two ways: from 12 to 12 h or 24 to 24 h, both for 60 h. Then, 12 h after the last treatment, the animals were euthanized, and the abdominal cavity was quickly opened, the colon removed, fragmented into similar sizes and opened for examination of macroscopic damage. Subsequently, sections were distributed for analysis.

### 4.10. Macroscopic Analysis

For the microscopic analysis, the animals were evaluated for intestinal damage by assigning a score using previously described a scale [62].

Score 0: No injuries.
Score 1: Hyperemia without ulceration.
Score 2: Linear ulceration without inflammation.
Score 3: Linear ulceration with inflammation.
Score 4: Two or more ulcerations and inflammation.
Score 5: Two or more ulcerations and inflammation or ulceration longer than 1 cm along the colon.
Score 6–10: If the lesions are greater than 2 cm in length longitudinally, 1 point for every extra inch will be awarded.

### 4.11. Assessment of Myeloperoxidase Activity

To evaluate the activity of MPO, colon samples from different groups were collected and frozen at −20 °C until use. After thawing, the samples were weighed and homogenized in 50 mM phosphate buffered saline (PBS), pH 7.4. The homogenates were centrifuged at 8000 rpm for 20 min at 4 °C. The pellets were resuspended in 50 mM PBS, pH 6.0, containing 0.5% hexadecyltrimethylammonium bromide (HETAB) and 10 M ethylenediaminetetraacetic acid (EDTA). The resulting homogenates were subjected to cycles of freezing/thawing and sonicated. To the homogenized sample (0.5 μL) was added 0.5 mL of solution containing 80 mM PBS, pH 5.4, 0.5% HETAB and 1.6 mM 3,3′,5,5′-tetramethylbenzidine (TMB). The mixture was incubated at 37 °C, and the reaction was initiated by the addition of 0.3 mM hydrogen peroxide. The readings will be held at 655 nm. One unit of MPO activity was defined as the amount of enzyme present that will produce a change in absorbance of 1.0 U/min at 37 °C in a final volume reaction containing the acetate. The results were quantified as mU/g of sample [63].

### 4.12. Evaluation of Alkaline Phosphatase Activity

In this model, alkaline phosphatase acts as the catalyst for the hydrolysis of nitrophenylphosphate sodium (5.5 mM) in glycine buffer (50 mM, pH = 10.5), which incorporates MgCl_2_ and forms a *p*-nitrophenol molecule, which presents a maximum absorption of 405 nm. The results were expressed as mU/mg of protein [64].

### 4.13. Nitric Oxide

The nitrite-nitrate concentration was measured using the Griess reaction and the addition of 100 μL samples of colon homogenized in 50 mM phosphate buffer, pH 7, obtained in item 2.8. Absorbance at 540 nm was measured using the ELISA reader.

### 4.14. Evaluation of Catalase Activity

Colon samples from different groups were used to verify the activity of catalase (CAT). Samples of colon were homogenized in 50 mM phosphate buffer, pH 7. In a cuvette, 2950 μL of the reaction solution (potassium phosphate buffer 50 mM, pH 7, and 20 mM hydrogen peroxide, 30 °C) was added to 50 μL of the diluted sample. The absorbance was measured at a wavelength of 240 nm for 5 min [65].

### 4.15. Analysis of Cytokines

Colon samples from the different groups were weighed and homogenized in 0.3 mL of the solution of phosphate buffered saline (PBS, pH 7.2) at 4 °C. Then, they were centrifuged at 4000 rpm for 5 min. The levels of IL-1 and IL-6 were determined using specific ELISA kits (enzyme immunosorbent assay), according to the manufacturer’s recommendations.

### 4.16. Histological Analyses

Colon samples from all groups were removed, fixed in formalin, embedded in paraffin and sectioned. The sections were flushed in hematoxylin and eosin.

### 4.17. Statistical Analyses

Values are presented as the mean ± standard deviation. Statistical analyses were done using Graphpad Prism. Analyses of variance (ANOVA) and Tukey-Kramer were used, considering *p* < 0.05 as statistically significant.

## 5. Conclusions

Extracts rich in glucans from the *Caripia montagnei* mushroom were used to evaluate their anti-inflammatory potential, well described in a model of colitis induced by TNBS. After the induction of colitis, the presence of colonic lesions, such as hyperemia and ulcerations, was observed. The results suggest that the increase in catalase activity by the polysaccharides of *C. montagnei* presents a protective effect in this model. Furthermore, their anti-inflammatory effect would be due to the decreased activity of myeloperoxidase and alkaline phosphatase, as well as the reduction of nitric oxide and IL-6, important inflammatory mediators of the inflamed colonic tissue, once again featured in the immunomodulating effects of this polysaccharide. Whether the anti-inflammatory action of *C. montagnei* in this model of colonic inflammation takes place exclusively by inhibition of inflammatory mediators, such as cytokines or NO, or by inhibition of the expression of NF-κB, still requires further investigation.
